# Supplementary material for: Lithium Chloride Protects against Sepsis-Induced Skeletal Muscle Atrophy and Cancer Cachexia
Source: Cells. 2021 Apr 26;10(5):1017. doi: 10.3390/cells10051017 (PMC8146089; doi:10.3390/cells10051017)
Supplement: Supplementary file 1 [file cells-10-01017-s001.zip › cells-1191821-SI.pdf]

Lithium chloride protects against sepsis-induced skeletal muscle atrophy and cancer cachexia

Ji-Hyung Lee[1], Seon-Wook Kim[1], Jun-Hyeong Kim[1], Hyun-Jun Kim[1], JungIn Um[1], Da-Woon Jung[1]* and Darren R. Williams[1]*

[1]New Drug Targets Laboratory, School of Life Sciences, Gwangju Institute of Science and Technology, 1 Oryong-Dong, Buk-Gu, Gwangju 61005, Republic of Korea

*To whom correspondence should be addressed: 1) Da-Woon Jung, School of Life Sciences, Gwangju Institute of Science and Technology, 1 Oryong-Dong, Buk-Gu, Gwangju 61005, Republic of Korea. Fax: Fax: +82 62-715-2484; Tel: +82-62-715-3554; email: jung@gist.ac.kr 2) Darren R. Williams, School of Life Sciences, Gwangju Institute of Science and Technology, 1 Oryong-Dong, Buk-Gu, Gwangju 61005, Republic of Korea. Fax: +82 62-715-2484; Tel: +82-62-715-2509; email: darren@gist.ac.kr

Supplementary figure S1

A

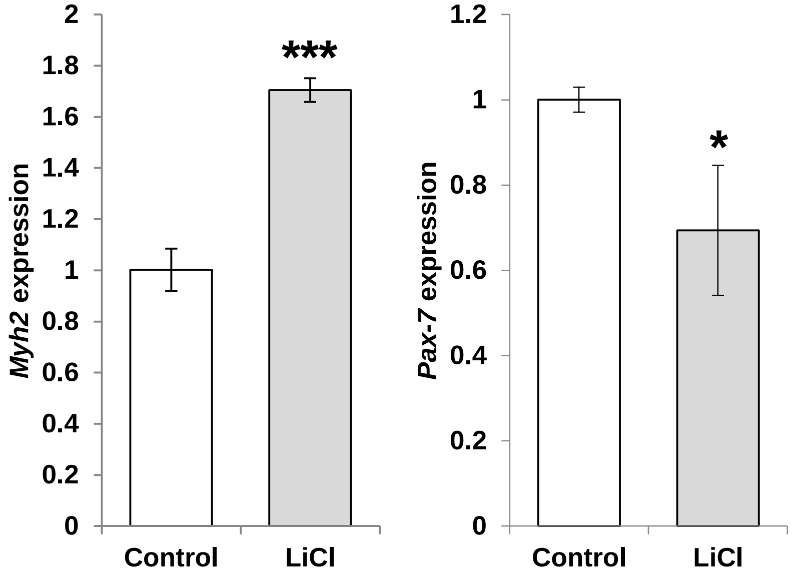

Figure S1. qPCR analysis targeting *Pax-7* and *Myh2* after 24 hours treatment of DM or DM containing 5 mM LiCl. Significant difference compared to control was marked * (*=*p*-value<0.05, ***=*p*-value<0.001)

Supplementary figure S2

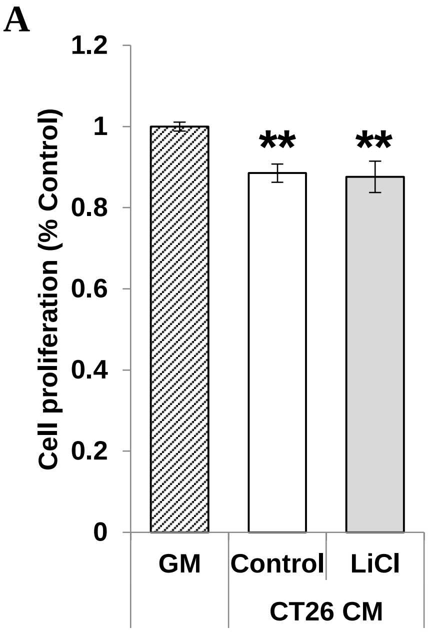

Figure S2. Cell proliferation assay for C2C12 myoblasts treated for 48 h with the indicated concentrations of LiCl (n=5). Significant difference compared to control was marked * (**=*p*-value<0.01)

Supplementary figure S3

A

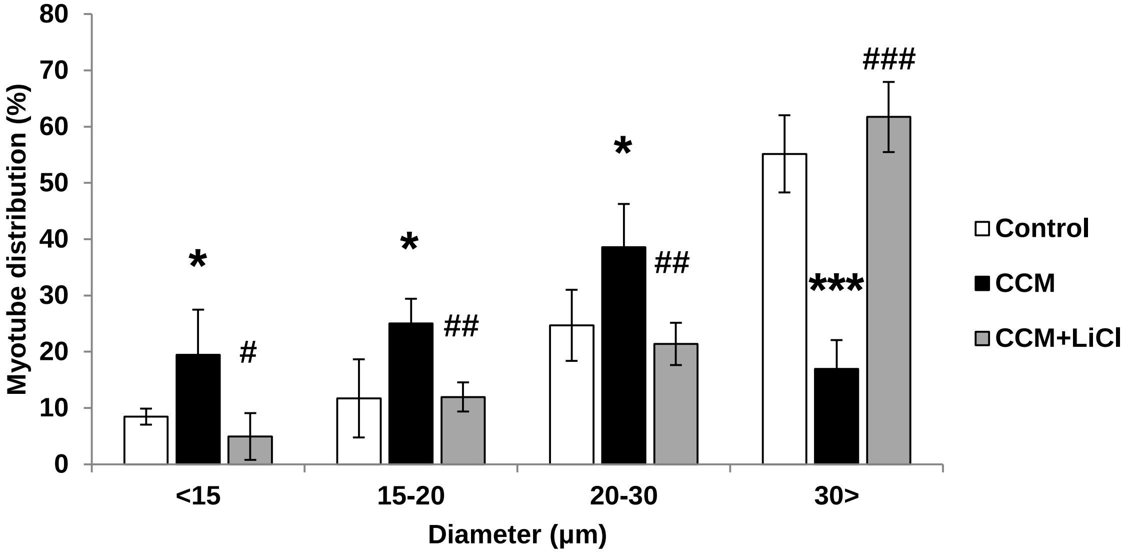

Figure S3. Frequency distribution of individual myotube diameters. Significance compared to DM treatment was marked with * (*=*p*-value<0.05, ***=*p*-value<0.001). Significance compared to CCM treatment was marked with # (#=p-value<0.05) ##=*p*-valve<0.01, ###=*p*-value<0.001).

Supplementary figure S4

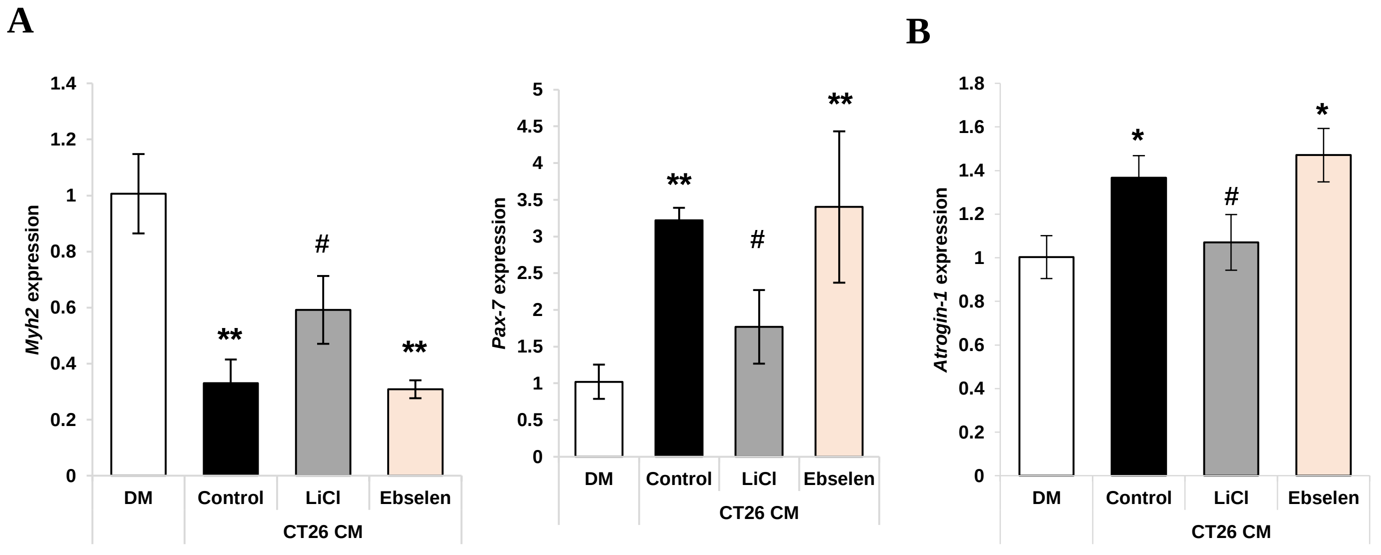

Figure S4. qPCR analysis for myotube marker, *Myh2*, and myoblast marker, *Pax-7*, after 24 treatment (n=3). (B) qPCR analysis *for Atrogin-1* after 24 h treatment (n=3). Significance compared to DM was marked with * (*=*p*-value<0.05, **=*p*-value<0.01). Significance compared to CT26 CM (CCM) treatment was marked with # (#=*p*-value<0.05).

Supplementary figure S5

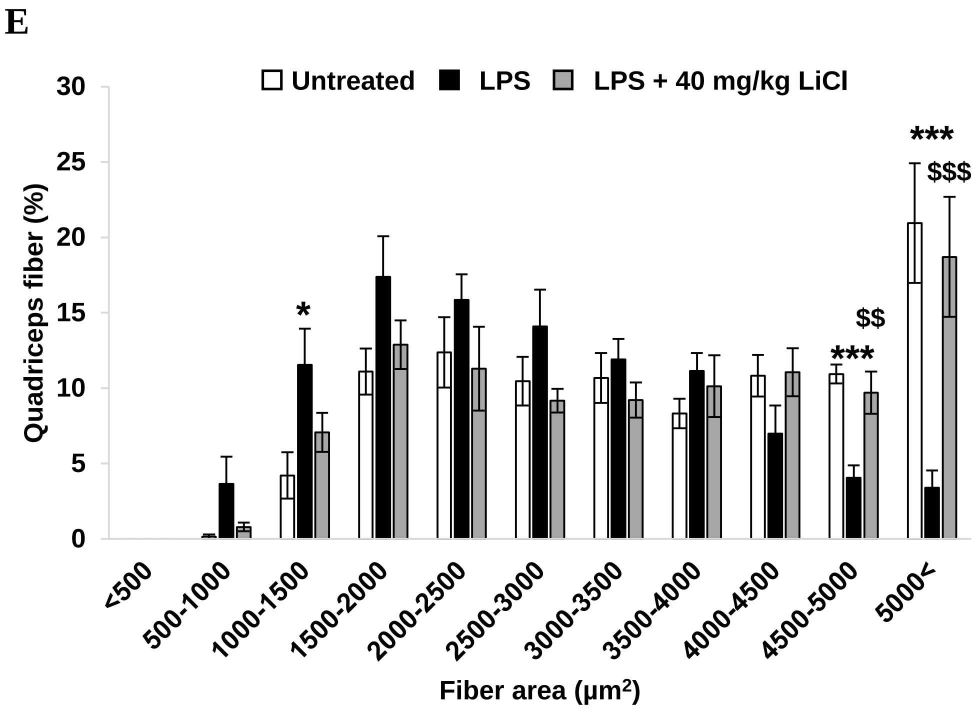

Figure S5. Frequency distribution of the cross sectional area in the quadriceps (n=5). Significant difference compared to untreated was marked with * (*=*p*-value<0.05, ***=*p*-value<0.001). Significant difference compared to saline plus LPS (control) was marked with $ ($$$=*p*-value<0.001).

Supplementary figure S6

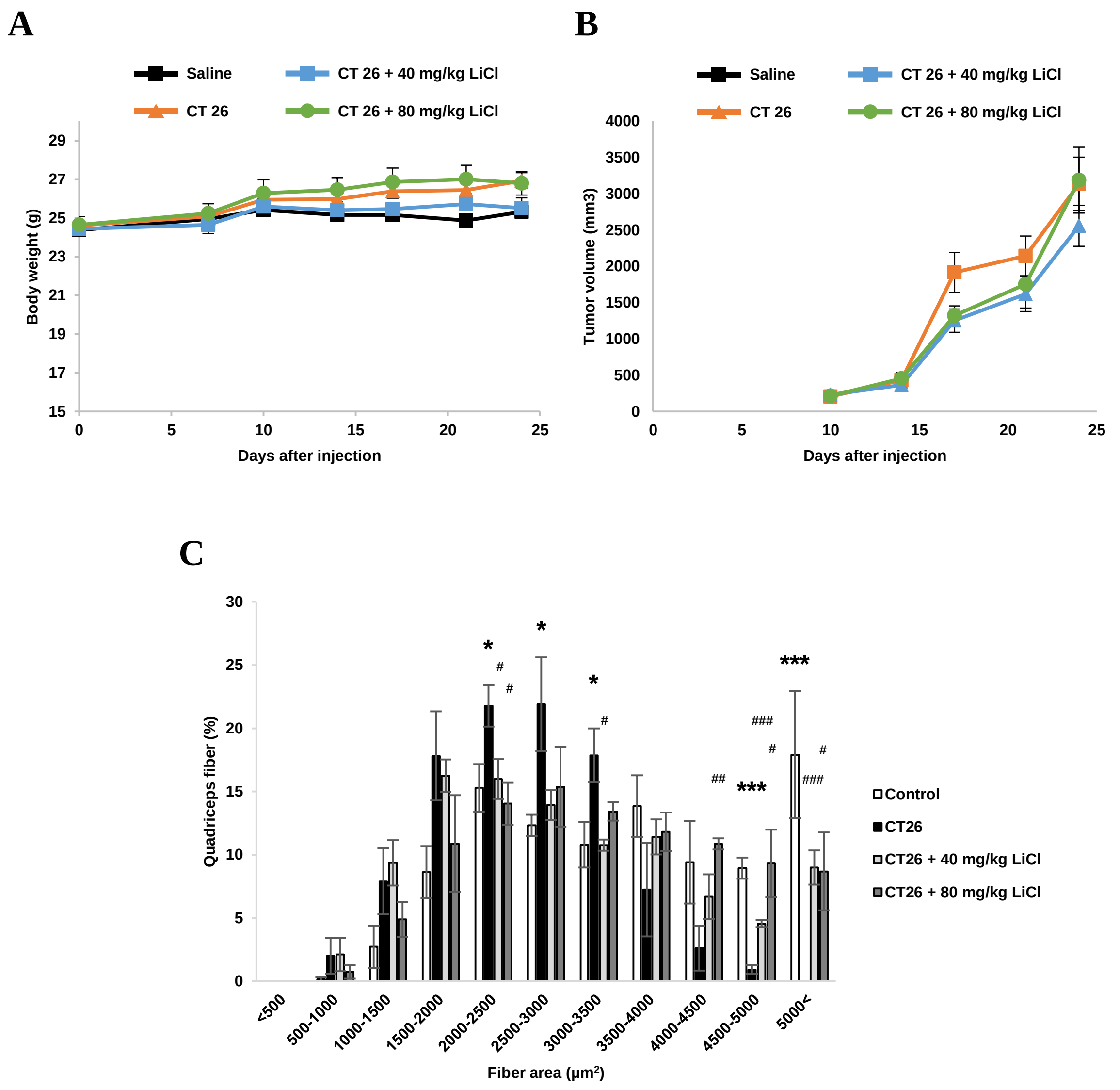

Figure S6. LiCl ameliorates muscle wasting in a mouse model of cancer cachexia. Mice were treated as follows: 1) no treatment, 2) transplantation with CT26 colon carcinoma cells, 3) transplantation with CT26 cells and treatment with 40 mg/kg LiCl, 4) transplantation with CT26 cells and treatment with 80 mg/kg LiCl. **(A)** Body weight change (n=5). **(B)** Time dependent growth of tumor volume (n=5). **(C)** Frequency distribution of the cross sectional area in the quadriceps (n=4). Significant difference compared to saline was marked with * (*=*p*-value<0.05, **=*p*-value<0.01, ***=*p*-value<0.001). Sig-nificant difference compared to CT26 control was marked with # (#=*p*-value<0.05, ##=*p*-value<0.01, ###=*p*-value<0.001).

Supplementary figure S7

For figure 1F Uncropped Western blot

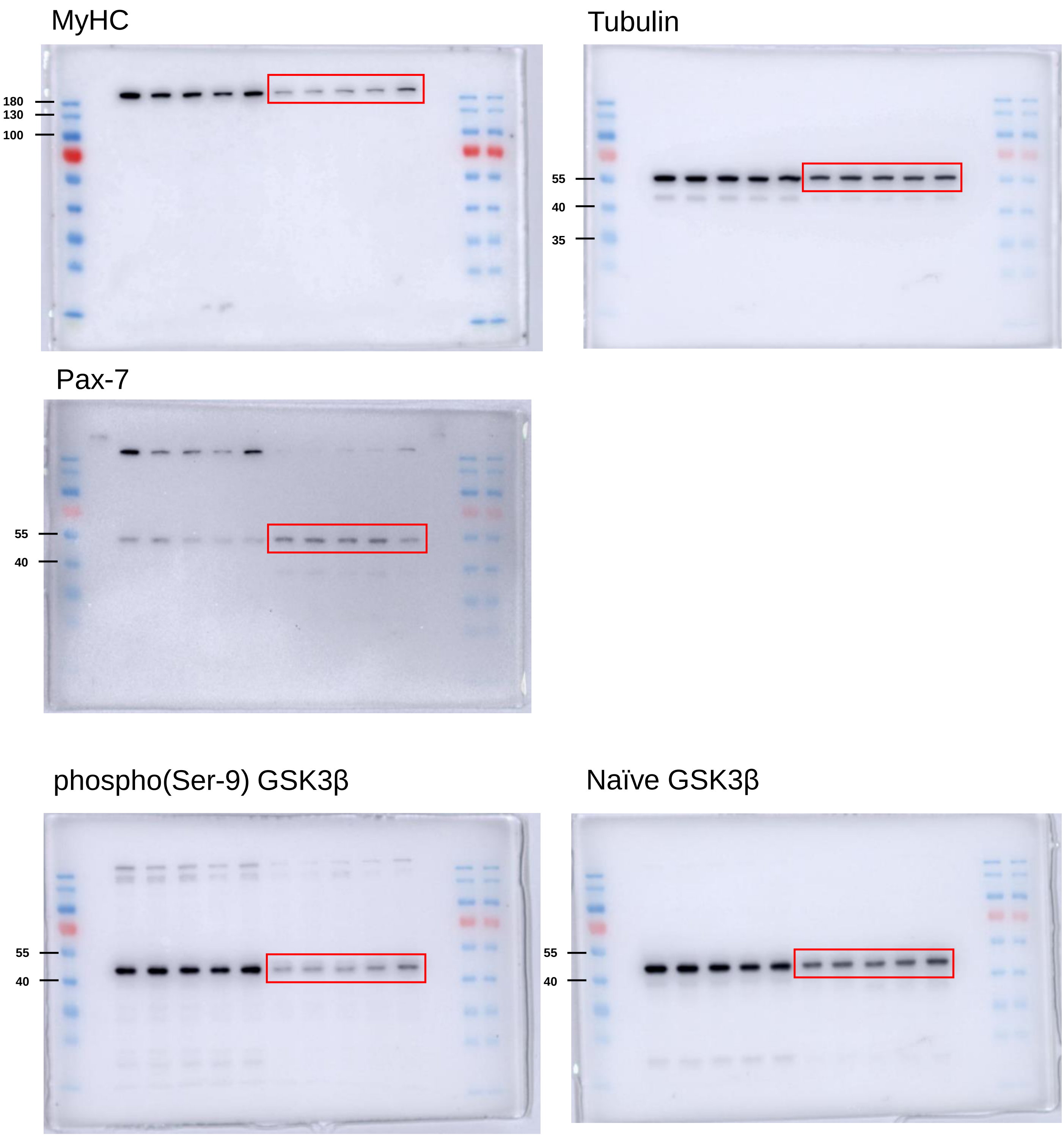

Figure S7 related to figure 1F. Uncropped western blot images for Figure 1F. Red boxes were cropped images for designated figures.

Supplementary figure S8

For figure 2C
Uncropped Western blot

MyHC

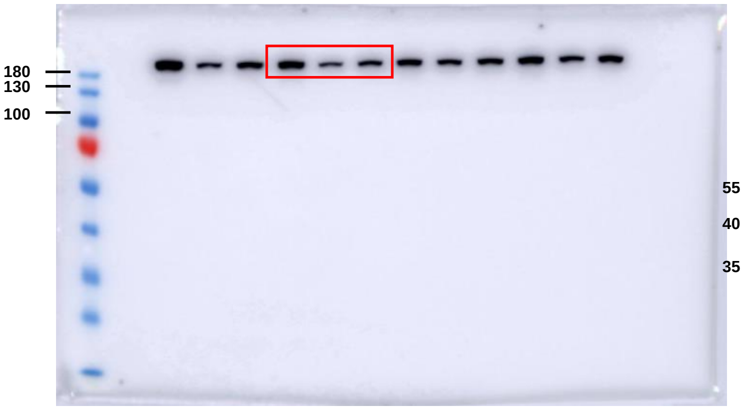

Tubulin

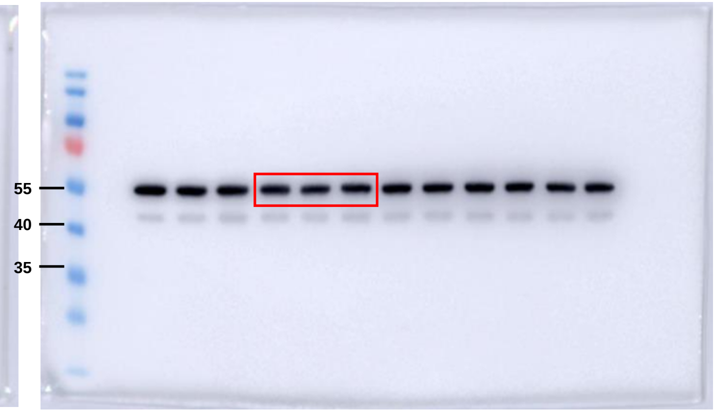

phospho(Ser-9) GSK3β

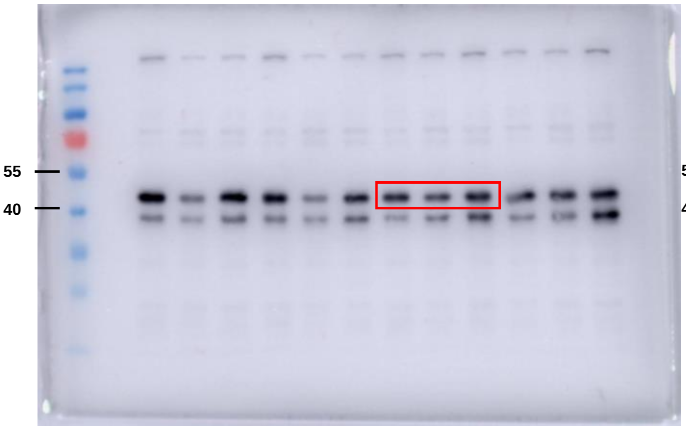

Naïve GSK3β

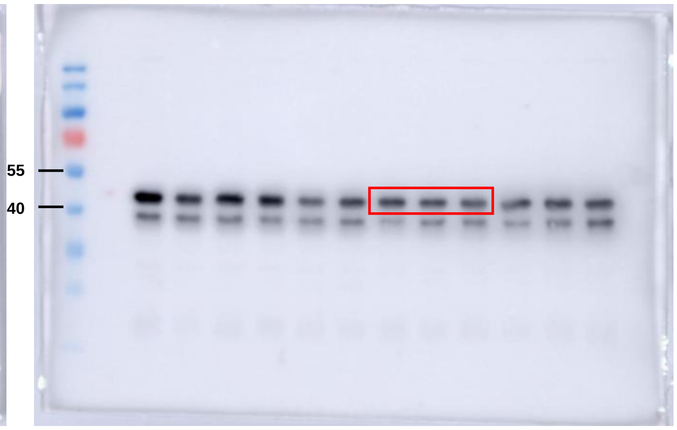

Figure S8 related to figure 2C. Uncropped western blot images for figure 2C. Red boxes were cropped images for designated figures.

Supplementary figure S9

For figure 3D Uncropped Western blot

MyHC

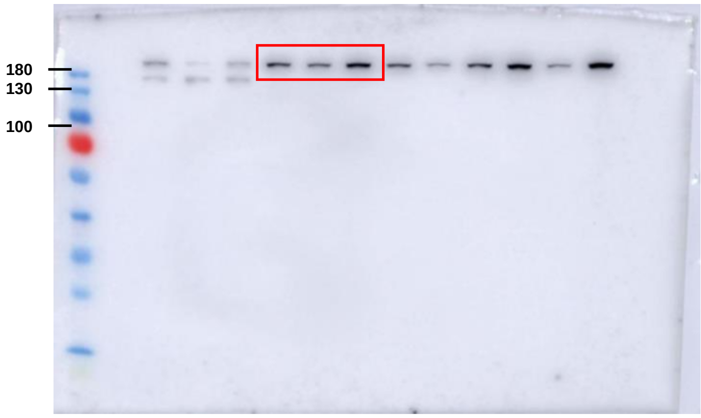

Tubulin

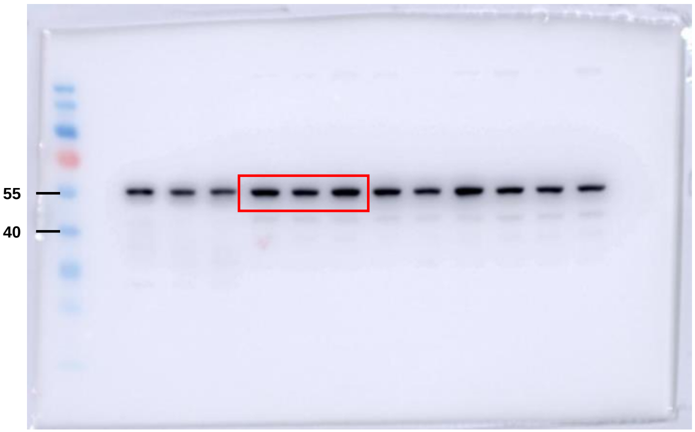

phospho(Ser-9) GSK3β

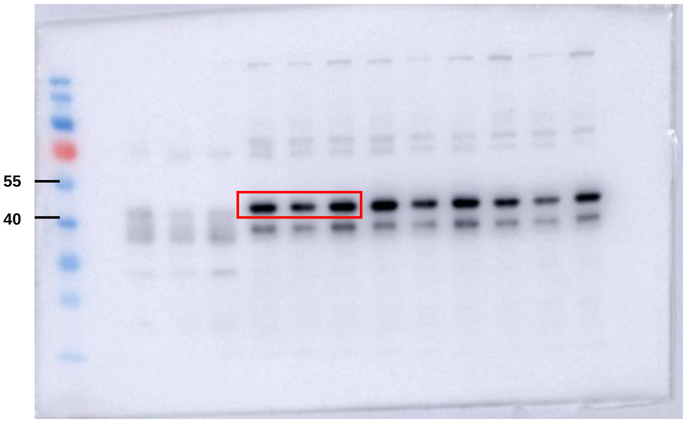

Naïve GSK3β

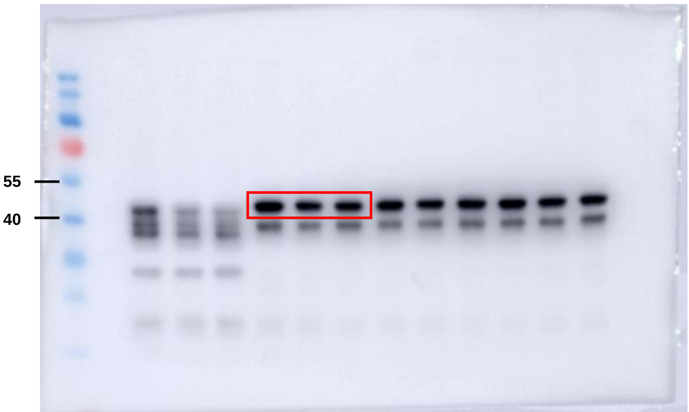

Atrogin-1

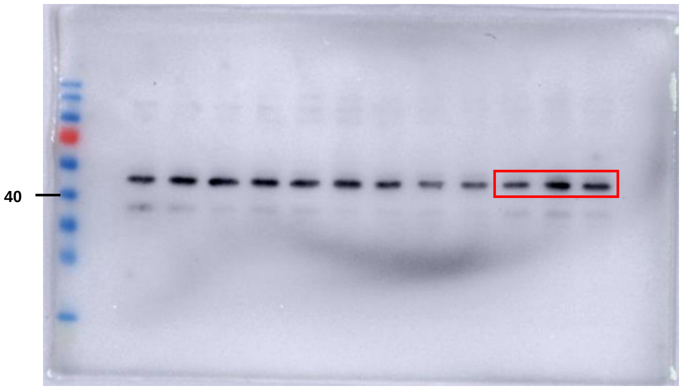

Tubulin

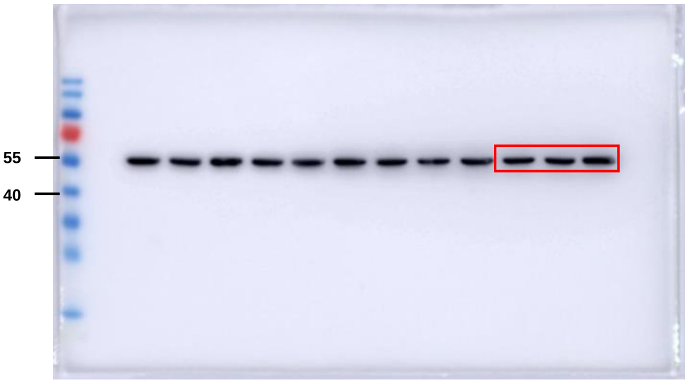

Figure S9 related to figure 3D. Uncropped western blot images for figure 3D. Red boxes were cropped images for designated figures.

Supplementary figure S10

For figure 4E Uncropped Western blot

IMPA1

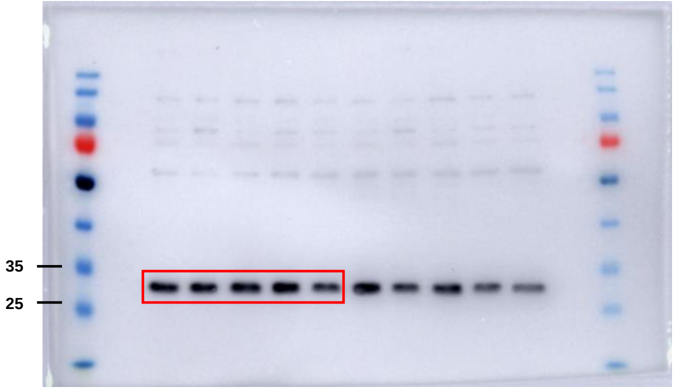

Tubulin

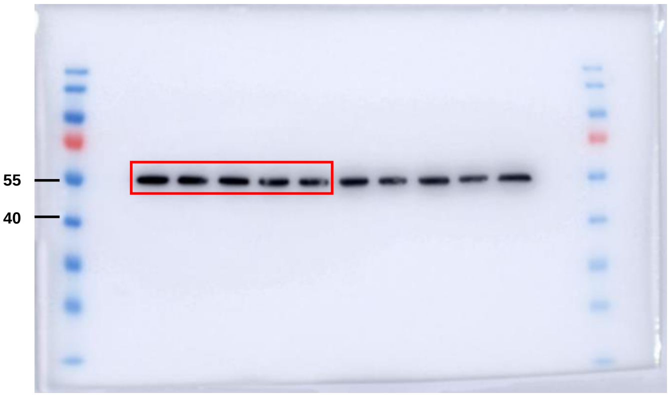

Figure S10 related to figure 4E. Uncropped western blot images for figure 4E. Red boxes were cropped images for designated figures.
